# Kartagener's syndrome: A rare condition diagnosed in a young male patient

**DOI:** 10.1016/j.radcr.2024.03.067

**Published:** 2024-04-19

**Authors:** Marina Balbino, Manuela Montatore, Federica Masino, Giuseppe Guglielmi

**Affiliations:** a Department of Clinical and Experimental Medicine, Foggia University School of Medicine, Viale L. Pinto 1, 71122 Foggia (FG), Italy; b Radiology Unit, “Dimiccoli” Hospital, Viale Ippocrate 15, 70051, Barletta (BT), Italy; c Radiology Unit, “IRCCS Casa Sollievo della Sofferenza” Hospital, Viale Cappuccini 1,71013 San Giovanni Rotondo (FG), Italy

**Keywords:** Kartagener's syndrome, Situs inversus totalis, Diagnostic imaging, Case report, Primary ciliary dyskinesia

## Abstract

Kartagener's Syndrome is a rare autosomal recessive genetic condition, that affects the structure and function of cilia and includes a condition of situs inversus, chronic sinusitis, and bronchiectasis associated sometimes with infertility.

A young patient who had a long-time fever, cough, and infertility after a clinical evaluation performed a chest X-ray and a CT scan that revealed the unexpected condition of Situs Inversus Totalis (SIT). Imaging also showed bronchiectasis and sinusitis: all findings consistent with Kartagener's syndrome, confirmed a second time by the genetic test.

This case highlights the importance of knowing and considering situs inversus in clinical practice, particularly when interpreting imaging studies and planning medical interventions. Furthermore, as situs inversus may be associated with cardiovascular and pulmonary pathologies in several syndromic conditions, such as Kartagener's syndrome in this case, these conditions should always be carefully examined.

## Introduction

Kartagener's syndrome (KS) is a rare autosomal recessive ciliary disorder caused by the condition of a primary ciliary dyskinesia; the primary issue is cilia movement defects, which cause repeated chest infections, ear/nose/throat problems, and infertility is very common in males; however, it may also be present in females, who may also experience ectopic pregnancies more often than normal.

The KS consist of a triad of situs inversus, bronchiectasis, and sinusitis [1], [2], [3], [4]. Situs inversus is a congenital condition characterized by a complete mirror-image reversal of the major visceral organs, where the organs are arranged in a pattern opposite to the normal positioning [5], [6].

Kartagener's syndrome also presents bronchiectasis, a condition of abnormal airways that results in chronic cough, sputum production, and respiratory impairment [7]. The severity of underlying bronchiectasis determines the radiological findings for Kartagener's syndrome in the chest. Findings that are visible on CXR and CT scans may include bronchial wall thickening and bronchial dilatation together with the absence of normal peripheral tapering. As supplementary discoveries, CT can also show a variety of other features, including consolidation regions, mucocele, tree-in-bud pattern, centrilobular nodules, and mosaic perfusion/air trapping.

## Case presentation

A patient, a 30-year-old non-smoker male, born to non-consanguineous parents came to the Emergency Department with a cough, shortness of breath, and long-time fever; he had also a history of recurrent respiratory infections.

The anamnesis of the patient revealed many a-specific symptoms such as frequently having colds, sneezing, and coughing with expectoration for the past 15 years, with shortness of breath, and despite the attempts he did not have children. The blood tests did not yield conclusive results, nor did they point toward a clear diagnosis. The family history was not pathologically relevant.

A cardiologist's clinical examination revealed dextrocardia and further imaging studies confirmed the diagnosis of situs inversus totalis [8].

**Fig. 1:**
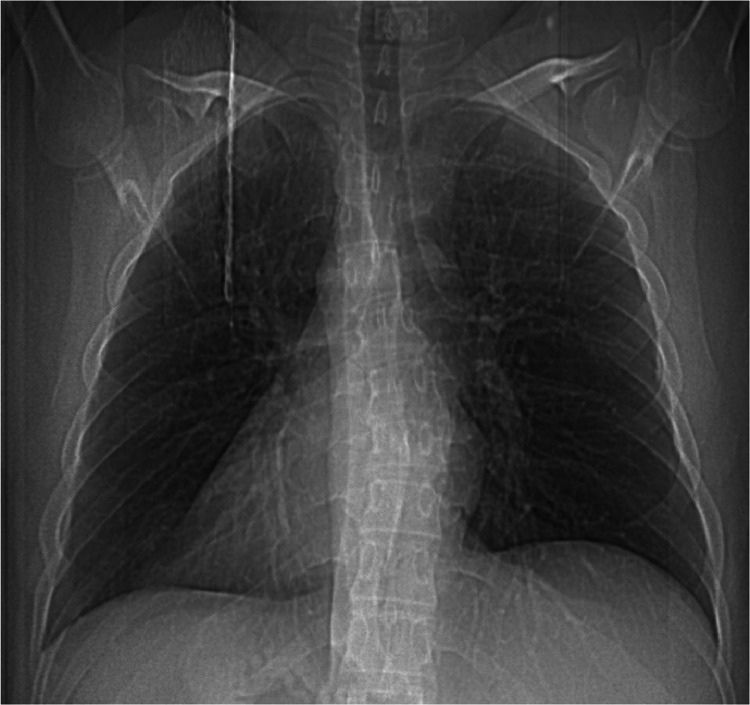
A chest X-ray showed a condition of dextrocardia and a condition of SI (Situs Inversus) in the chest. Due to this condition and the form of the rib's sinuses, a Situs Inversus Totalis (SIT) was suspected of involving the abdomen organs.

The diagnostic pathways after the clinical evaluation included: first a chest X-ray, a CT, and at the end, due to the suspect of the medical history, a genetic panel test which revealed and confirmed a mutation associated with Kartagener Syndrome. A chest X-ray of the patient was done (Fig. 1).

**Fig. 2:**
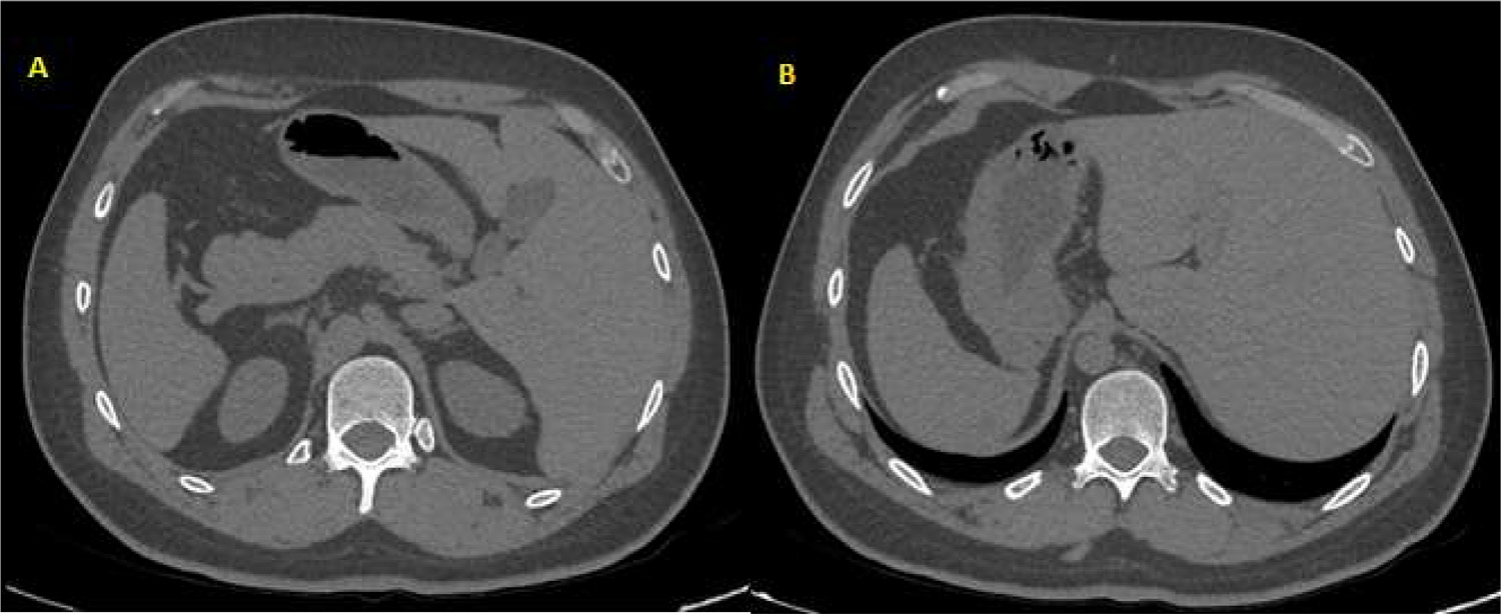
Axial CT image of the abdomen, without contrast medium at different levels. The anatomy of the abdomen was inverted: this is a condition of Situs Inversus Totalis (SIT). The stomach and spleen are on the left, and the bigger lobe of the liver is on the right.

At CXR there was a condition of dextrocardia. Due to the suspicion of a SIT a chest-abdomen CT scan was performed, which confirmed the inversion of the abdominal organs and confirmed the reversed position of the heart, previously displayed (Fig. 2).

**Fig. 3:**
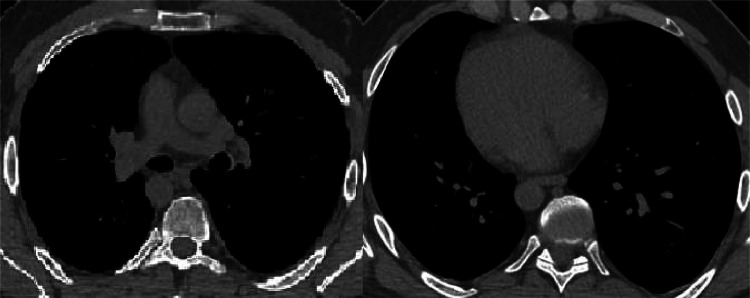
An axial CT image of the chest, without contrast medium confirmed the condition of dextrocardia, and the apex of the heart is on the right.

At the CT of the abdomen without contrast medium was discovered a SIT, with the opposed position of the abdominal organs. The CT of the chest and an echocardiography confirmed normal cardiac anatomy despite dextrocardia (Fig. 3).

**Fig. 4:**
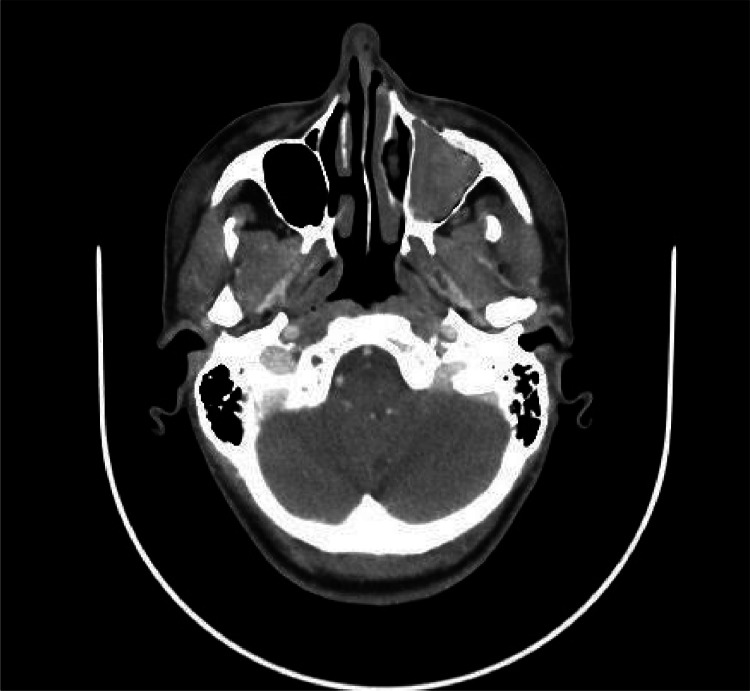
An axial CT image of the skull showed the left maxillary sinus obliterated.

At CT of the chest, there was no imaging evidence of bronchiectasis. Due to the lack of imaging evidence of bronchiectasis at the chest CT, which are part of the Syndromic panel, the radiologist asks for a strict follow-up of the thorax-imaging and to perform a bronchography; this exam excluded the presence of bronchiectasis. The diagnostic assessment was completed with the skull-CT that showed a condition of sinusitis (Fig. 4).

In the end the results of the genetic and other clinical tests confirmed, after some weeks, the diagnosis of a primary ciliary dyskinesia that, due to the syndromic symptoms as hypothesized with imaging, are called Kartagener's syndrome.

## Discussion

Kartagener's syndrome, also known as primary ciliary dyskinesia (PCD), is a rare genetic disorder that affects the structure and function of cilia [9]. Cilia are tiny, hair-like structures found on the surface of cells, particularly in the respiratory tract, reproductive organs, and other areas.

These cilia play a crucial role in moving mucus and other substances in a coordinated manner, helping to clear the airways and facilitate normal physiological processes [1], [2], [3], [4].

Features of Kartagener syndrome include situs inversus, chronic sinusitis, bronchiectasis, and male infertility. Situs inversus refers to a reversal positioning of the heart and major internal organs and it could be total (Situs Inversus Totalis) or incomplete (partial situs inversus) [5], [6].

It is referred to as a condition where only some visceral organs are transposed, while others remain in the normal position and are rare. It's important to note that while situs inversus can be associated with syndromes, not all individuals with situs inversus present an associated syndrome. The differential diagnosis was crucial in this clinical case, and of course, determining the reason for the cough and fever.

Primary ciliary dyskinesia is a rare genetic disorder characterized by recurrent respiratory tract infections, often associated with abnormal position of internal organs and, especially in males, infertility. The patient had opposed anatomy of the chest and the abdomen, clinical signs of pulmonary infection, sinusitis, and a personal story of infertility: the genetic test confirmed the condition of Katagener's syndrome. Mutations in genes coding for proteins involved in cilia shape and motility are the cause of the disease. Approximately 50 genes are known to be involved, but there are undoubtedly many more related to the disease that have yet to be identified. In general, primary ciliary dyskinesia is inherited in an autosomal recessive manner, which means that an individual must have mutations in both copies of one of the genes implicated to develop the disease. The altered genes are then inherited by the parents, who are usually healthy carriers.

For Kartagener's Syndrome, sometimes in addition to genetic testing which is, moreover, not always conclusive, the diagnosis makes use of a ciliary motility study and an electron microscopy examination of the cilia, from cells taken by brushing technique from the nose or bronchi.

Patients with Kartagener's syndrome often experience recurrent and chronic sinus infections due to impaired mucociliary clearance in the nasal passages. Bronchiectasis is the abnormal dilation of airways in the lungs [7].

The impaired ciliary function leads to difficulty clearing mucus, making individuals more prone to respiratory infections and bronchiectasis. Kartagener syndrome also can lead to male infertility due to impaired motility of sperm in the reproductive tract [10,11].

Diagnosis typically involves a combination of clinical evaluation, imaging studies, genetic testing, and specialized tests assessing ciliary function.

The interventions were focused on managing symptoms and preventing complications. The management plan of Kartagener's syndrome is often multidisciplinary, involving specialists in pulmonology, otolaryngology, and reproductive medicine, and is tailored to each patient's specific needs and may evolve based on the progression of the disease [10].

Some interventions could include bronchodilators, mucolytics, antibiotics, chest physiotherapy, and sinus surgery for respiratory symptoms. Assisted reproductive techniques, such as *in vitro* fertilization with sperm retrieval, may be considered for couples experiencing infertility due to immotile sperm [11]. Regular follow-up and communication with healthcare providers are essential for optimizing care and maintaining the best possible quality of life for individuals with Kartagener syndrome.

Educating patients about their condition, including the importance of adherence to medications and airway clearance techniques. It's also important to provide information about the genetic nature of the syndrome and its implications.

## Conclusion

Kartagener syndrome poses challenges; early diagnosis, comprehensive care, and ongoing research contribute to improving outcomes and enhancing the lives of individuals affected by this complex genetic disorder. Regular follow-up, especially long-term follow-up due to the young age of this patient, adherence to treatment plans, and awareness of potential complications are crucial for maintaining a good quality of life. Ongoing research aims to further understand the genetic basis and improve treatment options for the syndrome [12]. Advances in genetic therapies and targeted interventions hold promise for the future.

## Patient consent

Complete written informed consent was obtained from the patient for the publication of this study and accompanying images.

## References

[1] Mishra M, Kumar N, Jaiswal A, Verma AK, Kant S (2012). Kartagener's syndrome: a case series. Lung India.

[2] Liu BC, Huang TX, Liu CT (2020). Kartagener syndrome. Am J Med Sci.

[3] Barthwal MS (2006). Kartagener's syndrome in a fertile male–an uncommon variant. Lung India.

[4] Zurcher K, Kawashima A (2021). Kartagener's syndrome. N Engl J Med.

[5] Hernanz-Schulman M (1994). Situs inversus?. N Engl J Med.

[6] Spoon JM (2001). Situs inversus totalis. Neonatal Netw.

[7] O'Donnell AE (2022). Bronchiectasis - a clinical review. N Engl J Med.

[8] Chen XQ, Lin SJ, Wang JJ, Long S, Kong FX, Guo ZK (2022). Reverse life": a rare case report of situs inversus totalis combined with cardiac abnormalities in a young stroke. CNS Neurosci Ther.

[9] Gierich J, Otto J, Walt H, Dombi VH, Spycher MA (1997). Primäre ziliäre Dyskinesie mit Situs inversus ohne Bronchiektasen [Primary ciliary dyskinesia in situs inversus without bronchiectasis]. Pneumologie.

[10] Ibrahim R, Daood H (2021). Kartagener syndrome: a case report. Can J Respir Ther.

[11] Ceccaldi PF, Carré-Pigeon F, Youinou Y, Delépine B, Bryckaert PE, Harika G (2004). Syndrome de Kartagener et stérilité: observation, diagnostic et prise en charge [Kartagener’s syndrome and infertility: observation, diagnosis and treatment]. J Gynecol Obstet Biol Reprod (Paris).

[12] Gupta S, Handa KK, Kasliwal RR, Bajpai P (2013). A case of Kartagener's syndrome: importance of early diagnosis and treatment. Indian J Hum Genet.

